# “Time without you”: Transition to widowhood and its impact on time perspective and attitudes toward the future

**DOI:** 10.1007/s10433-025-00842-4

**Published:** 2025-02-13

**Authors:** M. Clara de Paula Couto, Yaeji Kim-Knauss, Klaus Rothermund, Helene H. Fung, Thomas M. Hess, Frieder R. Lang

**Affiliations:** 1 Institute of Psychology, Friedrich Schiller University Jena, Jena, Germany; 2 Institute of Psychogerontology, Friedrich-Alexander-University Erlangen-Nuremberg, Nuremberg, Germany; 3 Department of Psychology, Chinese University of Hong Kong, Shatin, Hong Kong; 4 Department of Psychology, North Carolina State University, Raleigh, USA

**Keywords:** Spousal loss, Critical life events, Time perspective, Subjective remaining life expectancy, Fixed-effects panel regression models

## Abstract

Widowhood is a significant life event that can profoundly alter an individual’s perception of time. Those who have lost a spouse often find themselves reflecting on past memories, while simultaneously feeling disconnected from the present. However, the impact of widowhood on one’s experience and perception of time has not been thoroughly explored. In this study, we investigate changes in time perspective during the transition to widowhood using a multidimensional approach to temporal experience. This perspective enriches the existing literature, which has primarily focused on the predictive role of time perspective by providing new insights into how major life events can reshape an individual’s experience of time. The sample was drawn from seven measurement points between 2009 and 2023 in the Aging-as-Future longitudinal study. It consisted of 1357 participants aged 50 and older who either remained married throughout the study period (*n* = 1270) or transitioned to widowhood (*n* = 87). We assessed four facets of time perspective: past-orientation, feelings of obsolescence, concreteness of the future time, and attitudes toward life’s finitude as well as subjective life expectancy. While the transition to widowhood predicted an increase in past-orientation and in feelings of obsolescence, it predicted a decrease in concreteness of the future. Spousal loss did not influence individuals’ attitudes toward finitude, but those experiencing widowhood reported a decrease in their perceived remaining lifetime. Our study showed that widowhood may lead to shifts in time perspective. Findings underscore the value of considering time perspective as a key indicator of an individual’s adjustment and functioning in response to a major life event.

## Introduction

The transition to widowhood is one of the most critical life events in adulthood that entails a period of intense suffering for most individuals. Consequently, this life event typically has negative effects on the widowed individual, such as increased depression (Lee et al. 2017; Schmitz 2021; Yoon et al. 2022), anxiety (Shear and Skritskaya 2012), and loneliness (Kapelle and Monden 2024). It also leads to decreased life satisfaction (Infurna et al. 2017; Lucas et al. 2003) and ultimately to an increased risk of mortality (Stroebe et al. 2007). A further dimension that may be affected by the loss of a spouse refers to how individuals experience time and think about their future (Brandtstädter and Wentura 1994). The loss of a spouse can profoundly affect how individuals experience and perceive different temporal dimensions: the past (i.e., when the spouse was still present), the present (i.e., shortly after the loss), and the future (i.e., in the absence of the spouse). For example, a widowed person may view the future as holding fewer opportunities while also feeling detached from the present. The past, on the other hand, may hold meaning, being a source of consolation in the good memories with one’s spouse. To the best of our knowledge, no previous studies have examined the consequences of widowhood on how individuals experience time and the future. In this study, we aim to examine the effect of widowhood on time perspective and attitudes toward the future.

### Widowhood and time perspective

Life circumstances or critical life events may deeply influence mental health and how individuals perceive and relate to time, affecting their connection to the past, present, and future (e.g., Åström et al 2019; Cleland et al. 2016). As highlighted by the Socioemotional Selectivity Theory (SST; Carstensen et al. 1999, for a recent review see Carstensen et al. 2024), the shrinkage of time horizons is associated with greater investments in a small circle of close, meaningful social ties, such as one’s spouse. For older adults, this means that marital ties become particularly important. Consequently, the loss of a spouse in old age can intensify the awareness of time without the spouse, leading to a reassessment of temporal perspectives. In the immediate aftermath of spousal loss, widowed individuals may experience a loss of the sense of meaning in life, often grappling with questions of identity and purpose (Koren and Lowenstein 2008). Moreover, the disruption caused by the loss of a spouse may extend to future aspirations and personal goals, which may suddenly seem unattainable or meaningless (Brandtstädter and Rothermund 2003; Brandtstädter et al. 2010). In response, individuals may need to adopt accommodative coping strategies that involve adjusting their goals to align with new realities and limited resources. The inability to find new sources of meaning and purpose can lead to feelings of alienation and disconnection from the present (Brandtstädter and Rothermund 2002; Brandtstädter et al. 2010). Thus, the transition to widowhood can significantly reshape an individual’s temporal outlook, which calls for more investigations.

Despite the destabilizing consequences of widowhood for individuals’ sense of purpose and goal setting, studies have largely overlooked the role of spousal loss and other critical life events in shaping how time is experienced. Instead, research has primarily focused on the predictive role of time perspective for other outcomes such as well-being (Kotter-Grühn and Smith 2011; Liao and Carstensen 2018), life satisfaction (Lu et al. 2018; Wirth et al. 2024), affective experiences and emotional functioning (Grühn et al. 2016; Katana et al. 2020), generativity (Hösch et al. 2023), negativity biases (Barber et al. 2020), depression (Åström et al 2019), or preoccupation with negative events (Strough et al. 2016). In other words, from the previous research, it becomes clear that time perspective is mainly understood as a predictor of psychological (mal)adjustment rather than an outcome (but cf. Brandtstädter and Wentura 1994). A recent systematic review by Kooij et al. (2018) examined the factors influencing future time perspective (FTP) and its consequences. The review emphasized that affective and personality traits—such as optimism, agreeableness, and openness—as well as agentic traits such as locus of control and self-efficacy, play a key role in shaping how individuals perceive their future. In addition, studies have conceptualized time perspective as a mediator highlighting its role as both an outcome and a predictor. For instance, research has shown that future time perspective (FTP) mediated the relationship between awareness of age-related losses and well-being (Brothers et al. 2016) and between age-related losses and depression (Dutt and Wahl 2019). Against this background, an examination of time perspective as an outcome of the transition to widowhood is still lacking. This study thus aims to address an important gap in the literature by investigating how widowhood affects individuals’ time perspectives.

### Time perspective models

Time perspective can be defined as the mental representation of past, present, and future time (Lewin 1942; see also Zimbardo and Boyd 1999). Relevant to the examination of time perspective as an outcome is to understand to what extent individuals experience time and the future as rigid or malleable. If the time perspective is rigid, then its role as an outcome variable is meaningless. In contrast, if the time perspective is malleable, it is plausible to assume that it differs depending on individual life circumstances.

Three models of time perspective can be differentiated regarding how time is mentally represented. Zimbardo’s time perspective model (Zimbardo and Boyd 1999) understands time perspective as a relatively stable, personality-like construct. In line with that perspective, there is no reason to expect that life events may alter time perspective. The SST (Carstensen et al. 1999) addresses the relevance of perceived time horizons (i.e., residual lifetime) for motivation, with changes in goal setting and future planning being associated with chronological age. Even though Carstensen et al. (1999) do assume that time perspective changes over the life span, their model focuses on future time perspective and its multiple components (e.g., global future horizons, future orientation and planning, and self-continuity; see Rutt and Löckenhoff 2016, see also Liao and Carstensen 2018), hence not considering other temporal frames. Like Carstensen et al. (1999), Brandtstädter and Wentura (1994) also examine time perspective from a developmental point of view. However, they propose a model of time perspective that includes multiple temporal frames such as the past, the present, and the future.

In line with Brandtstädter and Wentura (1994), at least four facets of time perspective can be defined: (1) *Past-Orientation* relates to the relative predominance of future- versus past-related content in people’s thoughts and perceptions of time, for example, whether individuals tend to think more often about their past life than their future. (2) *Feelings of Obsolescence* is a present-oriented facet of time perspective in that it reflects an individual’s alienation or detachment from the present time because of changes in social dynamics. (3) *Concreteness of the Future Time* refers to what extent individuals have clear and concrete ideas about their future and to what extent their planning and future horizon is structured in terms of content and time. Finally, (4) *Attitudes toward Life’s Finitude* deals with the individuals’ emotional attitude toward approaching the end of their life.

In their study on age-related changes in time perspective and attitudes toward the future, Brandtstädter and Wentura (1994) examined potential predictors of changes in time perspective (e.g., depression, coping styles, and health status), and found that beyond the fact that age was associated with changes in time perspective, depressive symptoms, for example, predicted lower levels of concreteness of the future time, higher levels of past-orientation, and higher levels of feelings of obsolescence. This study showed, therefore, that time perspective could indeed change as a consequence of life circumstances, which is crucial to our investigation of how the transition to widowhood impacts individuals’ experience of time and the future. In addition to that, a multidimensional model of time perspective is suitable for our aim to investigate the effects of widowhood not only in terms of future time but also in relation to how the past and present times are experienced as a result of changes in life circumstances.

### Current study

In this study, we draw from a sample of middle-aged and older adults to longitudinally examine how individuals experience time and the future in the transition to widowhood. We take a multidimensional time perspective that incorporates attitudes toward the past, the present, and the future. We thus aim to add to the current literature that has so far focused on the predictive role of time perspective, with a specific emphasis on future time. We investigate four facets of attitudes toward time and the future and propose the following hypotheses. Given the disruptive impact of widowhood on future-oriented plans and goals, we hypothesize that the transition to widowhood will be associated with lower levels of concreteness of the future time (*H1*). At the same time, levels of past-orientation will increase because the pre-loss, past time may be an important source of meaning for the widowed person (*H2*). The previous research discusses that reminiscence plays a significant role in coping with loss by facilitating the process of revisiting and recapturing meaningful memories from one’s past (Webster 1997). In terms of the experience of the present time, we hypothesize that in the transition to widowhood, individuals will feel alienated or detached from the present, leading to an increase in feelings of obsolescence (*H3*). We make no assumptions regarding the association between spousal loss and attitudes toward life’s finitude, as this facet of time perspective has been reported to become more accentuated in very old age when lifetime becomes reduced or when individuals are closer to the end of life (Brandtstädter et al. 2010; de Paula Couto et al. 2023; Lang and Rupprecht 2019). While age-related effects on attitudes toward life’s finitude have been reported, we found no prior empirical evidence regarding how, or whether, widowhood is specifically associated with this aspect of time perspective. Beyond these qualitative facets related to how individuals experience time and their future, we also examine the role of a more quantitative aspect of future time, which is known as subjective remaining life expectancy (SRLE). We hypothesize that the death of a spouse will increase the salience of mortality, leading people to perceive that they have fewer remaining life years (*H4*).

## Methods

### Participants and procedures

This study used secondary data from two subprojects of the Aging-as-Future study (AAF; Lang et al. 2024): the questionnaire study and the online study. Although overseen by different institutes with distinct thematic focuses, these two studies are connected through their membership in the same research consortium, ensuring regular collaboration, exchange, and methodological consistency.

Both studies included German, American, and Chinese (Hong Kong) samples as well as cross-sectional and longitudinal assessments spanning from 2009 to 2023, with time intervals that varied from 2 to 5 years between assessments. For the questionnaire study, three measurement occasions were available (2009, 2014, and 2019), whereas for the online study, six measurement occasions were available (2012, 2014, 2016, 2018/2019, 2020/2021, and 2023). For the current study, the final merged dataset included seven measurement points (2009, 2012, 2014, 2016, 2018/2019, 2020/2021, and 2023). German participants were recruited through local registry offices for the questionnaire study and via existing participant pools and public appeals (e.g., newspapers) for the online study. American participants were enlisted via field research companies, while Hong Kong participants were recruited through local contact points and a field research firm. The recruitment strategies for American and Hong Kong participants were consistent across both the questionnaire and online studies.

For the questionnaire study, participants answered the questionnaire alone, at home. After completing and returning the questionnaire, participants received a gift card valued at approximately $20 as compensation. Research procedures were approved by the Institutional Review Boards at Friedrich-Schiller-University Jena, North Carolina State University, and the Chinese University of Hong Kong. For the online study, participants who had previously consented received an email containing a link to a web-based questionnaire. While those who completed the questionnaire were remunerated approximately $20 up until the 2020 wave, no compensation was provided for the final survey in 2023. All data collection procedures adhered strictly to the ethical and data protection standards mandated by the Federal State of Bavaria, Germany, and by the Institutional Review Boards of the Chinese University of Hong Kong and North Carolina State University. More details on ethics approval, general sampling, and assessment procedure for both questionnaire and online studies are described in Lang et al. (2024).

To address our research questions, we focused on adults aged 50 and older who were married at baseline and either remained married throughout the study or transitioned to widowhood. As a result, we excluded participants who did not meet these criteria. We first refined the total available lifespan sample (*N* = 8828) to include participants aged 50 years and older who engaged in at least two time points (*n* = 2270). Within this subset, 1876 individuals retained a consistent marital status throughout the study period (*n*_consistent single_ = 127, *n*_consistent divorced_ = 239, *n*_consistent widowed_ = 178, and *n*_consistent married_ = 1332), while 321 individuals experienced changes in marital status during the study (*n*_single at baseline_ = 65, *n*_divorced at baseline_ = 75, *n*_widowed at baseline_ = 41, and *n*_married at baseline_ = 140). Additionally, there were individuals who reported marital statuses other than single, divorced, widowed, and married (*n*_other_ = 73). After removing duplicate entries where data were available from both questionnaire and online studies (*n*_duplicates_ = 117),^1^ we identified individuals who remained married throughout the assessment period (*n* = 1270 obtained from *n*_consistent married_ = 1332) and those who became widowed during the study (*n* = 87 obtained from *n*_married at baseline_ = 140), resulting in a final count of 1357 participants. This final subset represents 59.8% of the longitudinal sample aged 50 and above.

### Measures

#### Time perspective and attitudes toward the future

To evaluate the qualitative dimensions of time perspective, we used a set of items from the scales developed by Brandstädter and Wentura (1994), encompassing four facets: (1) concreteness of the future time (single item: “I believe that I still can achieve a lot in my life”), (2) past-orientation (the mean score of two items, “I more often think about my past life than the future” and “My thoughts often center on events that happened early in my life”), (3) feelings of obsolescence (the mean score of two items, “I find it increasingly difficult to cope with today’s way of life” and “I have increasingly less sympathy for the views of the younger generation.”), and (4) attitudes toward life’s finitude (the mean score of two items, “I look toward the end of life with calm” and “The thought of death burdens me”). Participants responded to all items on a 5-point Likert scale, ranging from “does not apply at all” to “completely applies.” McDonald’s omega—which is gaining preference due to its more realistic assumptions, such as the absence of tau equivalency (Malkewitz et al. 2023)—ranged from 0.72 to 0.84 across survey periods.^2^^,^^3^

#### Subjective remaining life expectancy (SRLE)

To measure future time perspective, we computed SRLE in years by subtracting participants’ calendar age from their expected life expectancy, as indicated by their response to the question “To what age do you expect to live?” (Kornadt et al. 2018; Kim-Knauss and Lang 2021). A higher SRLE denotes the perception of a greater number of years remaining in life.

#### Covariates

We controlled for the time-varying effects of subjective health and life satisfaction because (1) these variables were significantly correlated with all four facets of time perspective (see Table 2), and (2) they may reflect changes in physical and psychological health following spousal loss, which could influence shifts in time perspective (Brandtstädter and Wentura 1994; Wirth et al. 2024). Hence, including these variables as covariates allows us to better isolate the unique contribution of widowhood to changes in time perspectives. Subjective health was assessed by a single item, “How would you rate your current health?” in an ordinal scale ranging from 0 (“very poor”) to 4 (“very good”). For the analyses, subjective health was dummy-coded, using very poor health as reference category. Life satisfaction was assessed differently across the two subprojects included in this research. In the online subproject, life satisfaction was measured using an expanded scale ranging from 0 (“not at all satisfied”) to 10 (“completely satisfied”). In contrast, the questionnaire subproject employed a 5-point Likert scale. We thus computed t-scores for life satisfaction to adjust the mean and standard deviation of the two scales to be consistent.

### Analytical procedure

We employed fixed-effects panel regression models (FEM) to examine the impact of widowhood on changes in time perspective and attitudes toward the future. FEM enables us to estimate the relationship between variations in the explanatory variable (i.e., transition to widowhood) and changes in the outcome (i.e., time perspective). By utilizing each individual as their own control, FEM compares how individuals experience time and the future before and after experiencing widowhood. The average difference in outcomes pre- and post-widowhood thus yields an estimate of the “widowhood effect” on individuals’ perceptions of time and the future.

In contrast with cross-sectional analyses, FEM inherently adjusts for unobserved heterogeneity across individual variables that remain constant over time, such as chronological age at baseline, gender, or cultural background. This adjustment effectively eliminates the effects of these variables from the estimation (Wooldridge 2002). Hence, for the final model, we specified only time-varying variables, specifically marital status (0 = married and 1 = widowed), four dummy variables representing subjective health with very poor health as the reference point, and life satisfaction. These variables capture changes in their units over time, predicting the alterations in outcome variables.

We further carried out sensitivity analysis applying propensity score matching (Zhang et al. 2023). Prior studies have emphasized the existence of age-related differences on time perspective (Brandtstädter and Wentura 1994; Wirth et al 2024). Hence, by leveraging sensitivity analysis, we enhance the comparability between the married and widowed individuals, especially in relation to uncontrolled confounding effects of chronological age. Besides, the use of matching also addresses issues arising from the large disparities in the subsamples of married (*n* = 1270) and widowed participants (*n* = 87). The matched dataset for the sensitivity analysis comprised 86 widowed participants and 86 married participants, matched on baseline age, gender, and subjective health. One widowed case out of the total 87 could not be matched (1.1%).

## Results

### Descriptive analyses

The sample characteristics are presented in Table 1. We compared two groups depending on their marital status information, namely, the group “married” including those who are continuously married across all time points (*n* = 1270) and the group “widowed” comprising those who underwent the transition from married to widowhood during the survey times (*n* = 87).

**Table 1:** Demographic characteristics of participants at baseline by marital status

	Entire sample (*N* = 1357)		Matched sample (*n* = 172)	
	Married (*n* = 1270)	Widowed (*n* = 87)	Married (*n* = 86)	Widowed (*n* = 86)
	*M* (SD)	*M* (SD)	*M* (SD)	*M* (SD)
Age (in years)	64.19 (8.84)	71.32 (7.46)***	71.50 (7.68)	71.24 (7.47)
	Range: 50–87	Range: 52–84	Range: 52–87	Range: 52–84
Female, *n* (%)	575 (45.3)	57 (65.5)***	56 (65.1)	56 (65.1)
At least one child, *n* (%)	1137 (89.9)	80 (92.0)	75 (88.2)	79 (91.9)
Country, *n* (%)				
Germany	794 (62.5)	57 (65.5)	69 (80.2)	56 (65.1)
Hong Kong	324 (25.5)	20 (23.0)	8 (9.3)	20 (23.3)
USA	152 (12.0)	10 (11.5)	9 (10.5)	10 (11.6)
Subjective health	3.76 (0.91)**	3.43 (1.07)	3.53 (1.00)	3.44 (1.07)
Life satisfaction	51.97 (8.55)	50.24 (10.70)	53.20 (7.71)*	50.25 (10.77)
Dependent variables				
Concreteness of the future	3.23 (1.05)	3.08 (1.10)	2.80 (0.99)	3.10 (1.10)
Orientation to the past	2.80 (0.94)	2.88 (0.91)	2.97 (1.01)	2.88 (0.91)
Life’s finitude	3.67 (0.92)	3.74 (0.96)	3.63 (0.91)	3.74 (0.96)
Feelings of obsolescence	2.32 (0.90)	2.36 (0.91)	2.20 (0.77)	2.37 (0.92)
SRLE	19.72 (9.98)***	13.65 (7.98)	14.20 (7.68)	13.80 (7.92)

The significance signs denote the results of a *t*-test (for continuous variables) and a $x^{2}$-test (for categorical variables) comparing the two subsamples; SD, Standard deviation; SRLE, Subjective Remaining Life Expectancy; For Life Satisfaction, *T*-scores were computed to make the scales comparable; the group labeled “married” denotes individuals who remained married throughout the survey period, serving as control group; and the “widowed” group comprises individuals who underwent the transition to widowhood during the survey intervals

*p < .05; **p < .01; and ***p < .001

As shown in Table 1, in terms of baseline age, the widowed participants, with a mean age of 71 years, were significantly older than their married counterparts, whose mean age averaged around 64 years. Moreover, the widowed sample comprised a higher proportion of females (65.5%) compared to the married sample (45.1%), with this gender difference being statistically significant. Both samples had a large proportion of participants reporting having at least one child (89.2% for the married and 93.1% for the widowed), with no significant differences observed in this regard.

Our sample encompassed three distinct cultures: Germany, Hong Kong, and the USA. Most participants were based in Germany (62.5% for the married and 65.5% for the widowed), followed by Hong Kong (25.5% for the married and 23.0% for the widowed) and the USA (12.0% for the married and 11.5% for the widowed). However, no significant differences were found in terms of cultural proportions between the two samples. Regarding subjective health at baseline, the married group reported significantly better health compared to the widowed group. In terms of life satisfaction at baseline, both groups showed comparable levels.

Regarding our dependent variables at baseline, no significant differences were found in the qualitative facets of time perspective, including concreteness of the future time, past-orientation, attitude toward life’s finitude, and feelings of obsolescence. However, concerning subjective remaining life expectancy (SRLE), representing the quantitative aspect of future time perspective, and the widowed participants perceived significantly fewer years left in life compared to the married.

In terms of correlation patterns (see Table 2), past-orientation and feelings of obsolescence were similarly associated with subjective health and life satisfaction, with higher levels of past-orientation and of feelings of obsolescence being associated with worse subjective health and lower levels of life satisfaction. Additionally, past-orientation was associated with older baseline age, while feelings of obsolescence showed no such association. In contrast, higher levels of concreteness of the future time were associated with younger baseline age, better subjective health, and higher levels of life satisfaction. A more positive attitude toward life’s finitude was associated with older baseline age, better subjective health, and higher levels of life satisfaction. Finally, perceiving more remaining life years was only associated with younger baseline age and better subjective health.

**Table 2:**
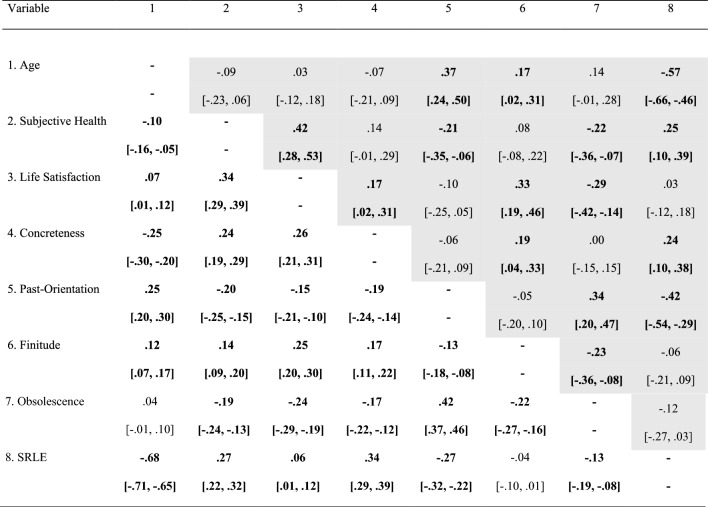
Correlation matrix at baseline

Statistically significant coefficients are printed bold (*p* < .05); the lower half indicates the correlation matrix of the entire sample (*N* = 1357), while the shaded upper half denotes that of the matched sample (*n* = 172); values in square brackets indicate the 95% CI for each correlation; Concreteness, Concreteness of the Future Time; Obsolescence, Feelings of Obsolescence; Finitude, Attitude toward life’s finitude; and SRLE, Subjective Remaining Life Expectancy

### Results from the fixed-effects panel regression

Table 3 displays the outcomes derived from the fixed-effects panel regression (FEM), elucidating alterations in various facets of time perspective after widowhood. The examined dimensions include concreteness of the future time, past-orientation, feelings of obsolescence, and attitude toward life’s finitude, representing qualitative aspects of time perspective and attitudes toward the future, while SRLE represents a more quantitative aspect of future time perspective.

**Table 3:** Fixed-effects linear regression models predicting changes in the future time perspective for the entire and the matched samples

	Entire sample (*N* = 1357)					Matched sample (*n* = 172)				
	CONC	PAST	FIN	OBS	SRLE	CONC	PAST	FIN	OBS	SRLE
	*B* (SE)	*B* (SE)	*B* (SE)	*B* (SE)	*B* (SE)	*B* (SE)	*B* (SE)	*B* (SE)	*B* (SE)	*B* (SE)
Widowhood (ref. married)	− 0.25**	0.29***	0.04	0.21**	− 2.98***	− 0.24*	0.29***	0.04	0.22**	− 3.04***
	(0.09)	(0.08)	(0.07)	(0.08)	(0.66)	(0.10)	(0.08)	(0.08)	(0.08)	(0.55)
Subjective health (ref. bad)	0.00	0.00	0.00	0.00	0.00	0.00	0.00	0.00	0.00	0.00
	(–)	(–)	(–)	(–)	(–)	(–)	(–)	(–)	(–)	(–)
Poor	− 0.12	− 0.12	− 0.03	− 0.25	0.73	− 0.06	− 0.07	− 0.50	− 0.00	− 1.09
	(0.16)	(0.14)	(0.13)	(0.14)	(1.17)	(0.34)	(0.26)	(0.26)	(0.28)	(1.81)
Fair	0.08	− 0.12	0.10	− 0.33*	1.89	0.22	− 0.14	− 0.32	− 0.01	0.20
	(0.16)	(0.14)	(0.13)	(0.14)	(1.17)	(0.35)	(0.27)	(0.27)	(0.28)	(1.86)
Good	0.19	− 0.17	0.10	− 0.32*	3.11**	0.36	− 0.21	− 0.20	0.11	1.28
	(0.17)	(0.14)	(0.13)	(0.14)	(1.19)	(0.37)	(0.28)	(0.28)	(0.30)	(1.96)
Very good	0.15	− 0.27	0.07	− 0.35*	2.97*	0.30	− 0.34	− 0.27	0.08	2.76
	(0.17)	(0.15)	(0.14)	(0.14)	(1.24)	(0.40)	(0.30)	(0.31)	(0.32)	(2.11)
Life satisfaction	0.01***	− 0.01**	0.01***	− 0.01**	0.03	− 0.00	− 0.01*	0.01*	− 0.00	0.01
	(0.00)	(0.00)	(0.00)	(0.00)	(0.02)	(0.01)	(0.00)	(0.00)	(0.00)	(0.03)
*R*-squared	0.02	0.02	0.02	0.01	0.03	0.04	0.08	0.05	0.03	0.14

CONC, Concreteness of the Future Time; PAST, Past-Orientation; OBS, Feelings of Obsolescence; FIN, Attitudes toward life’s finitude; and SRLE, Subjective Remaining Life Expectancy

*p < .05; **p < .01; ***p < .001

In support of our hypotheses, upon transitioning to widowhood from marriage (after controlling for individual time-constant variables, such as baseline age), individuals exhibited a decrease of 0.25 points in concreteness of the future (*H1*; *d* = − 0.10), an increase of 0.29 points in orientation to the past (*H2*; *d* = 0.15), and an increase of 0.21 points in feelings of obsolescence over time (*H3*; *d* = 0.10). Conversely, the transition to widowhood did not influence individuals’ attitudes toward finitude. Supporting *H4*, those experiencing widowhood reported a decrease of 2.98 years in SRLE in comparison with the pre-loss period (*d* = − 0.23), indicating a decrease in perceived remaining lifetime after transitioning to widowhood. In supplementary multilevel models that explicitly included between-person control variables available in our dataset (i.e., calendar age, gender, and the presence of children), the main findings remained consistent, except for the concreteness of the future model, where the significance level became marginal. Nonetheless, FEM remains the optimal primary analytic approach because it is particularly robust against unobserved, between-person confounding variables that could potentially influence pre-existing differences in widowhood and time perspectives, which our dataset could not directly measure.

Concerning the influence of subjective health, as a time-varying covariate, significant effects appeared only in models with feelings of obsolescence and SRLE as outcomes. Findings revealed that individuals reporting improved health over time, transitioning from very poor to either fair, good, or very good perceived health, experienced a decrease in feelings of obsolescence. Regarding SRLE, individuals whose subjective health improved from bad to good or very good reported a significant increase in SRLE over time, indicative of an increase in perceived remaining lifetime.

In terms of life satisfaction's influence, significant effects were observed in the models assessing the qualitative aspects of attitudes toward the future. Individuals who reported a one-unit increase in life satisfaction (*T*-scores) over time reported a corresponding increase of 0.01 points both in concreteness of the future and in finitude. However, they experienced an equal decrease of 0.01 points in orientation to the past and in feelings of obsolescence over time.

### Propensity score matching analysis

Corroborating that the sample for the sensitivity analysis was properly matched, we found no statistically significant differences in the baseline characteristics between the matched married and widowed participants (see Table 1).

Besides, no differences were found between the matched married and widowed participants in terms of culture, subjective health, and dependent variables at baseline. Most importantly, when applying the matched data to FEM, all main effects of widowhood on time perspective and attitudes toward the future time remained robust (see Table 3), with increased effect sizes (*d* = − 0.15 for concreteness of the future; *d* = 0.40 for orientation to the past; *d* = − 0.30 for feelings of obsolescence; and *d* = − 0.60 for SRLE).

## Discussion

Using longitudinal data from the AAF study, we examined the effects of widowhood on individuals’ experience of time and the future, taking a multidimensional temporal approach. By investigating time perspective as an outcome of widowhood and considering the multidimensionality of time (i.e., past, present, and future), our research contributes to understanding the widowhood effect in terms of time perspective. The main findings of the study indicated a clear pattern according to which the transition to widowhood negatively impacted the time perspective with (1) decreasing levels of concreteness of the future time and the life years that were perceived to be left in life and (2) increasing levels of feelings of obsolescence and of past-orientation, which are present- and past-related facets of time. Decreases in subjective remaining life expectancy may indicate that spousal loss may have made the end of life more salient for the widowed individuals. The previous research has shown that individuals who perceive fewer remaining years tend to cope less effectively with losses and critical life events (Rothermund and Brandtstädter 1998). Therefore, our finding that perceived remaining life expectancy decreases following spousal loss may be particularly concerning for adjustment to widowhood.

Decreases in the concreteness of the future and increases in feelings of obsolescence may signal a loss of important sources of meaning. In contrast, a shift toward a more past-oriented perspective reflects a focus on sources of meaning derived from an individual’s biography—sources that tend to gain importance with age (Brandtstädter et al. 2003). After the loss of a spouse, these biographical sources may become particularly significant. However, adjusting to the loss of a loved one likely involves not only reconfiguring the bond with the deceased but also adapting to the present. This aligns with Rubin et al. (2020) two-track model of bereavement, which proposes that succesful adaptation to loss involves both maintaining attachment to the deceased and adjusting to life in the present.

Our findings align with and further extend the perspective that widowhood is a major critical life event with significant negative impacts on mental health (e.g., increased depression and anxiety; see Schmitz 2021, and Shear and Skritskaya 2012, respectively), life satisfaction (e.g., Infurna et al. 2017), and social integration (e.g., loneliness; Kapelle and Monden 2024). If the death of a significant attachment figure puts widowed individuals at risk of developing a wide range of difficulties, most bereaved individuals navigate their loss without experiencing long-term issues (Rubin et al. 2020). Regarding long-term effects of widowhood, information on the time elapsed since widowhood was not available in the AAF dataset. Prior research has shown that the effects of widowhood on psychological outcomes can persist for up to 4 years following the spousal loss (Lucas et al. 2003; Lin and Brown 2020; Streeter 2019). The varying time intervals in our dataset likely capture widowhood effects spanning from the immediate aftermath to approximately 4 years post-loss, consistent with this critical timeframe when the widowhood effect is still salient.

Nonetheless, due to the varying time intervals in our dataset and the limited availability of precise information on the timing of widowhood, we were unable to explicitly investigate the effects of time elapsed since widowhood in more detail. To address this, we conducted multilevel analyses that controlled for the study type (i.e., questionnaire vs. online), as time interval differences were primarily confounded with this factor. These analyses showed no significant changes in the main findings, further confirming the robustness of our results despite this limitation.

Additionally, we tested whether baseline levels of our outcome variables differed between individuals who entered the study already widowed (and were thus excluded from the analysis) and those who entered as married but later experienced widowhood (the focal group of this study). Interestingly, no significant differences were found in the baseline levels of time perspective variables. This suggests that the observed changes in time perspective may not be long-lasting after widowhood. These findings align with prior research on recovery trajectories following widowhood (Lucas et al. 2003) and highlight the need for future studies using more precise temporal data to better understand how the timing of widowhood influences time perspectives.

Past research has discussed the role of different personal and social circumstances surrounding the experience of widowhood. For example, regarding the cause of death for the deceased partner—whether sudden or resulting from a prolonged illness—could moderate the impact of spousal loss on time perspective. Additionally, among spousal caregivers, aspects such as caregiver stress and caregiving demands are associated with time constraints, physical and mental exhaustion (see, e.g., Keene and Prokos 2008). In line with this, the “relief hypothesis” suggests that the end of spousal caregiving through widowhood may mitigate the negative effects of spousal loss (Keene and Prokos 2008; Schaan 2013).

Marital quality may function as another moderator of the effects of widowhood, with prior studies showing that individuals who report higher satisfaction with their marriage experience higher levels of psychological distress during the transition to widowhood as compared to individuals who report lower marital quality (Carr et al. 2000; Schaan 2013). Finally, the negative effects of widowhood for mental health and well-being have been shown to be moderated by gender with findings mostly showing that widowhood takes a heavier toll on widowers who take longer to adapt to spousal loss than widows (e.g., Streeter 2019; Yoon et al. 2022). Investigating the moderating role of additional variables in the association between widowhood and change in time perspective would be important for future studies. Our sample of widowed individuals was, however, not large enough for such analyses. These questions thus must be delegated to future research.

It is noteworthy that our main findings regarding the impact of widowhood on changes in time perspectives were adjusted for the influence of culture. While our study did not specifically examine cross-cultural differences in how widowhood affected time perspective, it is worth considering whether our findings can be generalized across different cultural contexts in the future research. Despite the lack of extensive literature on this topic, widowhood may elicit a universal psychological and emotional response, at least in the short term. Thus, potential cultural variations, if any, might rather confound with individual factors, such as family structure, which could be particularly less or more prevalent in certain cultures (Jadhav and Weir 2018). Further exploration in this area is warranted to better understand these associations.

The influence of human development to the representation of past, present, and future has been previously reported (e.g., Brandtstädter and Rothermund 2003; Brandtstädter et al. 2010; Carstensen et al. 1999). In line with that idea, throughout life the focus on the future decreases, while the present and the past gain more relevance. Our sample includes married and widowed individuals 50 years and older, and the widowed sample was older than the married sample at baseline. Our model controls for baseline age; furthermore, to account for any uncontrollable confounds related to age, we carried out a propensity score match analysis that clearly showed that the obtained effects with the entire sample remained significant even when having matched samples that did not differ in their age, gender, and subjective health.

### Limitations

This study has several strengths, including a longitudinal design and a multidimensional assessment of time perspective. However, there are also limitations to consider. First, our study involves a relatively small sample widowed individuals (*n* = 87) compared to those who remained consistently married throughout the study (*n* = 1270), which could limit the generalizability of the findings. To address this issue and optimize comparability, we performed propensity score matching by selecting a subset of 86 individuals from the consistently married group, matched on age, gender, and subjective health. This allowed for a more balanced comparison between the two groups while retaining the unique strengths of our dataset. Future research with larger and more balanced sample sizes is needed to further validate and extend this study findings.

We have singularly investigated the impact of widowhood on how individuals experience time and their future. In adulthood, however, different stressful life events may occur that bring about negative consequences for those who experience such events (e.g., decreased health and well-being, see Cleland et al. 2016; de Paula Couto et al. 2011). Hence, it would be important for future studies to examine whether time perspective is affected by other stressful life events that are often reported in adulthood, which may disrupt personal beliefs and sense of meaning, such as health deterioration, house move, divorce/marital dissolution, death of a child or a parent, and unemployment.

In our study, we have not investigated length of widowhood, in other words, the time elapsed since spousal loss (e.g., Koren and Lowenstein 2008; Yoon et al. 2022). Past research has shown that the negative effects of widowhood, such as higher levels of depressive symptoms (Yoon et al. 2022) and loss of meaning (Koren and Lowenstein 2008), tend to be more pronounced in the short-term (i.e., during the reaction phase, see, e.g., Lucas et al. 2003), starting in the 1st year following spousal loss and usually lasting approximately 2–4 years (Lin and Brown 2020; Streeter 2019). Since we did not ask participants to specify the year of spousal loss, the time elapsed since widowhood could not be explicitly included in this study.

Lastly, while the internal consistency of the employed subscales was acceptable, as indicated by the Spearman–Brown coefficients and McDonald’s omega, we acknowledge the limitations of relying on a reduced set of items from the Time Perspective and Attitudes toward the Future Scale (Brandtstädter and Wentura 1994). For example, our measure of the concreteness of the future relied on a single item (“I believe that I still can achieve a lot in my life”), which, while capturing a general sense of future-oriented optimism, does not encompass the full multidimensionality of the original construct. The original item set for that facet includes aspects such as specific goal setting, vividness of envisioned futures, and emotional components such as worries and confidence. These dimensions are particularly relevant in understanding how spousal loss affects an individual’s capacity for planning and engaging with the future. Given that widowhood can profoundly alter future aspirations and personal goals (Brandtstädter and Rothermund 2003; Brandtstädter et al. 2010), future research should aim to incorporate these omitted dimensions.

## Conclusion

Replicating and extending previous research that reported the negative effects of experiencing widowhood for a broad range of psychological variables, our study showed that the transition to widowhood further impacted how widowed individuals experienced time and their future. Therefore, this study contributed to addressing three important gaps in the literature: First, our findings indicated that time perspective changed as a consequence of critical life events; second, the use of a multidimensional approach to time perspective has proven to be relevant as the direction of changes in time perspective depended on specific temporal frames; and third, the study was the first to test the effects of widowhood specifically on time perspective, providing evidence that spousal loss negatively impacts the experience of time and future perspective.

Our study has important practical implications considering that time perspectives can influence a range of health behaviors such as taking medication, seeking care, doing advance care planning, and even financial behaviors like saving versus spending. Research has shown that perceiving the future as limited is associated with lower engagement in healthy behaviors, such as physical activity (Stahl and Patrick 2012) and maintaining a healthy diet (Gellert et al. 2012). Many of these health-related activities are often easier to engage in with a partner, a support system that widowed individuals lack. As such, both the loss of a spouse and particular types of time orientation, like feeling disconnected from the present or perceiving the immediate or distant future as meaningless, may undermine the individual’s motivations to prepare for a healthy future, which is a key determinant of functioning and well-being in later life (Kornadt et al. 2019; Wirth et al. 2024). Additionally, our findings may inform interventions aimed at supporting individuals during the bereavement process. For example, incorporating both past-oriented (reminiscing) and present-oriented (adjusting to the present loss) aspects of time perspective—referred to as the two tracks of bereavement (Rubin et al. 2020)—could help widowers navigate the grieving process. Such an approach might enhance engagement in health behaviors and life planning by reducing feelings of obsolescence, improving future envisioning, and mitigating a sense of limited life expectancy.

## Data Availability

The raw data supporting the conclusions of this article will be made available by the authors upon request. The R code for the main analyses is available in the following: https://osf.io/mdn3v/?view_only=3312fc01aaed4674b994bb96c7f8e3e6.

## Footnotes

1 In cases of such duplicate data, we only retained the online part of the study, as it provided a greater number of available time points for analysis.

2 Given that each facet was examined as a separate outcome, we conducted the Spearman–Brown coefficient test to assess the reliability of subscales comprising two items: past-orientation, feelings of obsolescence, and attitudes toward life’s finitude. The Spearman–Brown coefficients demonstrated adequate reliability, with past-orientation ranging from .61 to .69, feelings of obsolescence from .50 to .67, and attitudes toward life’s finitude from .51 to .76.

3 To examine potential differences in how the measurement was understood across study modes (i.e., questionnaire and online), we tested measurement invariance (MI) by fitting a three-factor model encompassing past-orientation, feelings of obsolescence, and attitudes toward life’s finitude (i.e., the facets consisting of more than one item). This analysis utilized data from two waves where both modes were available (2014 and 2018/2019). In 2014, we found evidence of metric invariance, indicating that participants interpreted and responded to the items similarly across both modes. However, in 2018/2019, partial invariance was observed, requiring one item from the attitudes toward life’s finitude subscale to be freed. We in fact observed slight differences in the Spearman–Brown coefficients for the attitudes toward life’s finitude subscale in the 2018/2019 wave by study types: .55 for the questionnaire study and .46 for the online study. While the findings generally support comparability, such slight variances highlight the need to consider potential confounding effects of study type when interpreting our findings.
